# α-Aryl substituted GdDOTA derivatives, the perfect contrast agents for MRI?†

**DOI:** 10.1039/d3cc05989h

**Published:** 2024-01-09

**Authors:** Karley B. Maier, Lauren N. Rust, Fabio Carniato, Mauro Botta, Mark Woods

**Affiliations:** a Department of Chemistry, Portland State University 1719 SW 10th Ave Portland OR 97201 USA mark.woods@pdx.edu; b Dipartimento di Scienze e Innovazione Tecnologica, Università del Piemonte Orientale “Amedeo Avogadro” Alessandria I-15121 Italy mauro.botta@uniupo.it; c Advanced Imaging Research Center, Oregon Health and Science University 1381 SW Sam Jackson Park Road Portland OR 97239 USA woodsmar@ohsu.edu

## Abstract

Enhancing the performance of Gd^3+^ chelates as relaxation agents for MRI has the potential to lower doses, improving safety and mitigating the environmental impact on our surface waters. More than three decades of research into manipulating the properties of Gd^3+^ have failed to develop a chelate that simultaneously optimizes all relevant parameters and affords maximal relaxivity. Introducing aryl substituents into the α-position of the pendant arms of a GdDOTA chelate affords chelates that, for the first time, simultaneously optimize all physico-chemical properties. Slowing tumbling by binding to human serum albumin affords a relaxivity of 110 ± 5 mM^−1^ s^−1^, close to the maximum possible. As discrete chelates, these α-aryl substituted GdDOTA chelates exhibit relaxivities that are 2–3 times higher than those of currently used agents, even at the higher fields (1.5 & 3.0 T) used in modern clinical MRI.

## Abstract 2

Introducing aryl substituents into the α-position of the well-known GdDOTA affords chelates that have unprecedentedly high relaxivities and robustness.

## Graphical abstract

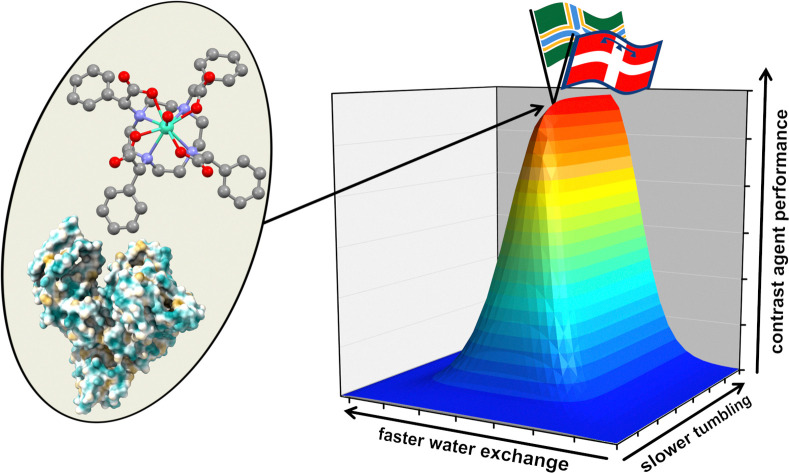

Over recent years two major challenges have emerged to the use of Gd^3+^ chelates as contrast agents in magnetic resonance imaging (MRI). The emergence in the early 2000s of nephrogenic systemic fibrosis (NSF) in patients with compromised renal function who had undergone contrast-enhanced MRIs raised concerns over the safety of Gd^3+^ chelates.^1,2^ Although regulations limiting contrast agent use in renally deficient patients have eliminated NSF, these safety concerns seem to have lingered.^3^ The use of contrast agents in MRI is also causing pollution. Normally locked in the lithosphere, Gd^3+^ levels in some parts of the hydrosphere are now substantially higher than geogenic levels.^4,5^ Gd^3+^ is detectable in some drinking water sources^6,7^ as well as in marine life.^8,9^ The long-term impacts of this pollution are unclear but, coupled with the lingering concerns over Gd^3+^ safety, this problem needs to be addressed.

A common solution can be applied to both these problems: reduce the amount of Gd^3+^ administered for an MRI exam while retaining its diagnostic efficacy. The Gd^3+^ chelates used as MRI contrast agents are notoriously inefficient (Fig. 1),^10^ requiring high doses: typically 1.0–1.5 g per dose. Theory shows that the performance of a Gd^3+^ chelate as a relaxation agent can be substantially improved. The relaxivity of a chelate depends upon the number of solvent water molecules that can coordinated directly to the Gd^3+^ (*q*), the distance of their protons from the metal (*r*_GdH_), the rate at which they exchange with the bulk solvent (1/*τ*_M_) as well as the rate at which the chelate tumbles (1/*τ*_R_) and the electronic relaxation parameters *Δ*^2^ and *τ*_V_. Fig. 1 shows that the contrast agents currently in use are sub-optimal: exchanging water too slowly and tumbling too rapidly.^11^ There is significant scope to improve the performance of Gd^3+^ chelates and thereby facilitate a dose reduction that would reduce risk and surface water pollution.

**Fig. 1:**
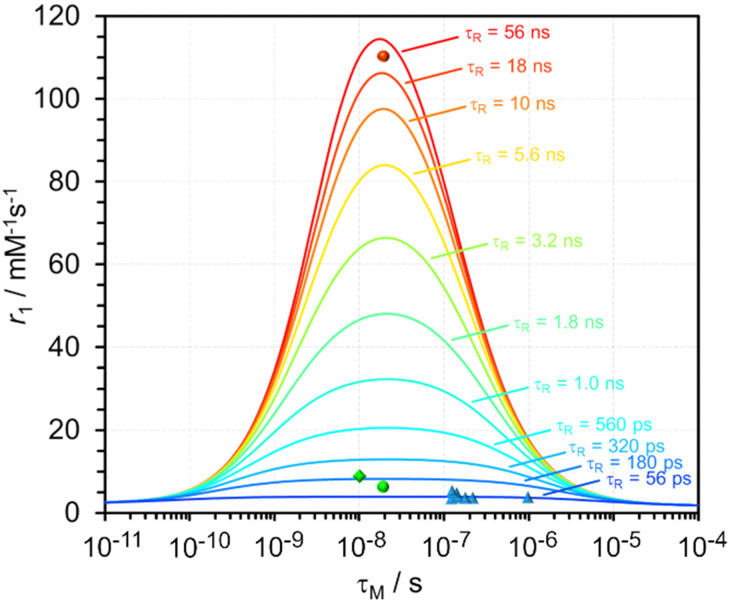
The calculated relaxivity (the increase in water proton relaxation rate constant per mM Gd^3+^) of a Gd^3+^ chelate that has one site open for coordination by water, as a function of the water exchange lifetime (*τ*_M_) for different rotational correlation time constants (*τ*_R_). In this calculation the *B*_0_ field is 0.5 T, other values were fixed to: *r*_GdH_ = 3.0 Å, *Δ*^2^ = 6.8 × 10^−18^ s^−2^ and *τ*_V_ = 25 ps. The relative positions of some clinical agents (blue triangles), GdDOTFA (green circle), GdDOTBA (green diamond) at 310 K and GdDOTFA bound to human serum albumin, assuming a 1-to-1 binding model, (red circle) at 298 K are shown.

The choice of ligand framework is an important consideration for a Gd^3+^ chelate that may be used *in vivo*. The emergence of NSF demonstrated the importance of resistance to Gd^3+^ dissociation and thus the use of macrocyclic ligands.^1,12^ Ligands based around the common DOTA framework are therefore good candidates, but GdDOTA itself has sub-optimal water exchange kinetics.^13^DOTA chelates are found to adopt both square antiprismatic (SAP) and twisted square antiprismatic (TSAP) coordination geometries. This is of significance because water exchange in TSAP isomers is found to be up to 100× faster than the SAP isomer.^14–16^GdDOTA predominates as the SAP isomer, increasing the proportion of TSAP isomer is a strategy for improving water exchange kinetics.^16,17^ It is known that substituting the α-position of the pendant arms of a DOTA chelate will increase the proportion of TSAP isomer present.^15,17,18^ We recently reported an efficient method of introducing aryl substituents into the α-position of DOTA chelates (Fig. 2).^19^ This substitution was found to increase the TSAP/SAP ratio (298 K) from 1 : 4 (EuDOTA) to 7 : 1 (EuDOTFA) and 10 : 1 (EuDOTBA). This change in isomeric ratio was found to have a profound effect on increasing the rate of water exchange. Analyzing the Gd^3+^ chelates by variable temperature ^17^O NMR afforded the water exchange lifetimes: *τ*_M_ = 19 ns (GdDOTFA) and *τ*_M_ = 10 ns (GdDOTBA) (Fig. S1, ESI†). These α-aryl substituted DOTA chelates exhibit water exchange that are optimal for achieving the highest relaxivities (Fig. 1).§§GdDOTFA and GdDOTBA were prepared using previously described methods.^19^ The relaxometric analyses described herein were performed using the same instrumentation and methods described previously.^28^

**Fig. 2:**
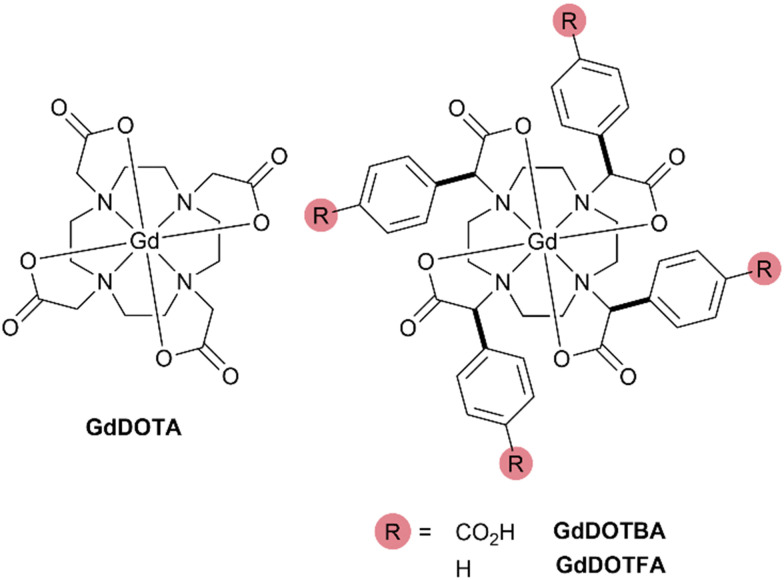
The structures of the Gd^3+^ chelates of DOTA, DOTFA and DOTBA.

Extremely high relaxivities were not expected for either GdDOTFA or GdDOTBA because these chelates are comparatively low molecular weight and will tumble quickly in solution. Nonetheless, a good deal of information about these chelates can be obtained from a quantitative analysis of the nuclear magnetic relaxation dispersion (NMRD profiles) of these chelates which measure relaxivity as a function of the applied magnetic field (*B*_0_). The NMRD profiles at 298 K of both GdDOTFA and GdDOTBA (Fig. 3) are notable for several reasons. The relaxivity at all fields is significantly higher than that of GdDOTA, this can be attributed to the larger size of these chelates – slower rotational tumbling (longer *τ*_R_). At low fields this difference is especially pronounced: indicative of more favourable electronic relaxation properties. This is confirmed by the results of simultaneously fitting the NMRD profiles with the VT ^17^O NMR data (Table 1). The relationship between coordination chemistry and electronic relaxation is not entirely clear but the aromatic substituents appear to have the effect of shielding the chelate from collision, which reduces the rate of modulation of the zero-field splitting (ZFS). Additionally, the square of the trace of the ZFS tensor (*Δ*^2^) is significantly smaller than in most DOTA type chelates. Together these have the potential to lift the limiting effect of electronic relaxation on relaxivity typically observed for DOTA type chelates.

**Fig. 3:**
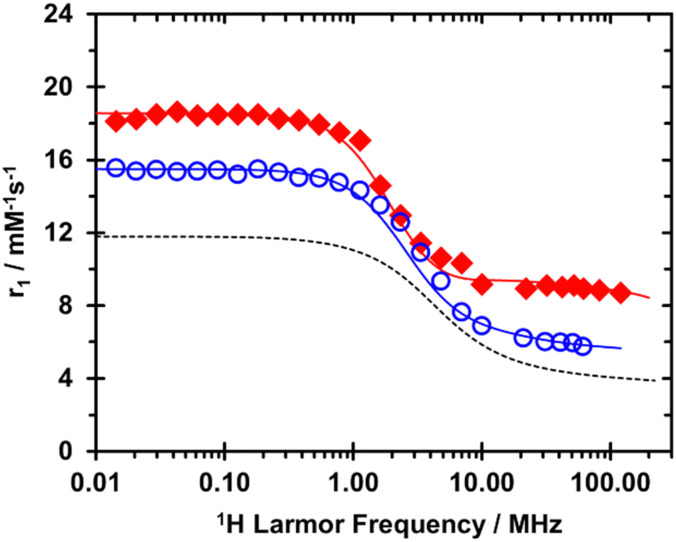
^1^H NMRD profiles, recorded at 298 K, of GdDOTFA (open blue circles) and GdDOTBA (closed red diamonds). For reference the (298 K) ^1^H NMRD profile of GdDOTA is shown (dashed line).

**Table 1:** Selected fitting parameters for the ^17^O VT NMR and ^1^H NMRD profiles of GdDOTFA and GdDOTBA. Parameters fixed during fittings: *r*_GdH_ = 3.0 Å, ^298^*D* = 2.24 × 10^5^ cm^2^ s^−1^, *a* = 4.0 Å, *A*_O_/*ħ* = −3.6 × 10^6^ rad s^−1^

Parameter	GdDOTFA	GdDOTBA	GdDOTA^a^
^0.5^ *r* ^298^_1_/mM^−1^ s^−1^	8.1	11.4	4.8
*A*_O_/*ħ* 10^6^ rad s^−1^	−3.6 ± 0.1	−3.6 ± 0.2	−3.7
*τ* ^298^_M_/ns	19 ± 0.6	10 ± 1	261
*τ* ^298^_R_/ps	161 ± 10	274 ± 7	66
*Δ* ^2^/10^18^ s^−2^	6.8 ± 0.2	10.0 ± 0.4	16
*τ* ^298^_V_/ps	25 ± 2	31 ± 1	7.7

a From ref. 13.

To achieve the highest relaxivities from a Gd^3+^ chelate it is necessary to make a substantial reduction in the rate of tumbling.^20,21^ One commonly employed strategy for achieving this goal is binding to a macromolecule such as a protein. Human serum albumin (HSA) is a commonly used protein for this purpose because a simple hydrophobic interaction can be used to couple the motion of the chelate to that of the protein.^22^GdDOTFA and GdDOTBA both have aromatic substituents with the potential to bind to HSA. Accordingly, HSA was titrated into solutions of GdDOTFA and GdDOTBA and binding was monitored by relaxometry at 0.5 T and 298 K (Fig. S2, ESI†). Fitting these data using a simple 1 : 1 binding model shows that the binding of both chelates to HSA was quite weak. GdDOTBA binds to HSA more strongly (*K*_a_ = 1143 ± 117 M^−1^) than GdDOTFA (*K*_a_ = 220 ± 15 M^−1^). Perhaps the stronger association of GdDOTBA with the protein arises from the fact that this chelate is more negatively charged, enhancing the interaction of the chelate with positively charged residues around the binding site.^23^ Given the differences in binding constants, it may reasonably be supposed that the nature of the interaction with HSA is different for each of these two chelates. This may give rise to differences in other factors such as chelate orientation and freedom of the chelate to rotate locally, each of which may impact relaxivity.^22^ The relaxivity of GdDOTBA is found to increase by 521% upon binding: the relaxivity of the chelate when bound to HSA: *r*^bound^_1_ = 59 ± 3 mM^−1^ s^−1^ at 20 MHz and 298 K. This significant improvement in relaxivity is modest in comparison to the 1361% increase measured for HSA-bound GdDOTFA: *r*^bound^_1_ = 110 ± 5 mM^−1^ s^−1^ at 20 MHz and 298 K! Even though the association between GdDOTFA and HSA is weak. To our knowledge this is the first discrete *q* = 1 Gd^3+^ chelate to achieve theoretical maximal relaxivity (Fig. 1).^22^ The calculation of maximal relaxivity stipulates that the chelate contains a single Gd^3+^ ion and has just one open binding site for coordination by water. No Gd^3+^ chelates that meet these criteria have previously attained the highest relaxivity allowed for by theory. Such high relaxivity suggests that DOTFA binds to HSA in a way that effectively couples the motion of the chelate to that of the protein,^24^ it also indicates the value of optimizing of several key parameters: *τ*_M_, *Δ*^2^ and *τ*_V_.

Increasing the effective molecular mass of a Gd^3+^ chelate sufficiently to maximize relaxivity will cause an agent to extravasate and excrete more slowly. This reduces its utility as a contrast agent and potentially recreates the safety concerns of NSF. To replace the current crop of contrast agents with new ones that can perform equally well at lower doses will require lower molecular weight chelates. In terms of performance as a low molecular weight contrast agent, GdDOTBA is worthy of further examination.

At higher *B*_0_GdDOTA exhibits the slow, steady decrease in relaxivity generally observed for Gd^3+^ chelates as the magnetic field increases. GdDOTFA exhibits a similar decrease. But in the NMRD profile of GdDOTBA there is a small relaxivity “hump” centred around 1 T. This is indicative of a more slowly tumbling chelate, but this hump is small. And because water exchange in GdDOTBA is very fast, this hump is pushed to higher fields than usual (0.5 T is typical) (Fig. S4 and S5, ESI†). The result of these considerations is that the relaxivity of GdDOTBA is both quite high and almost unchanged over the range *B*_0_ = 0.5–3.0 T. At 298 K the relaxivity at 3.0 T is 11.35 mM^−1^ s^−1^ which compares favourably with 11.7 mM^−1^ s^−1^ at 1.5 T and 11.4 mM^−1^ s^−1^ at 0.5 T.

The relaxivity of GdDOTBA compares very favourably with established clinical contrast agents^25^ – between 2 and 3 times higher (ESI† and Fig. 4, left). Only two more recent entries – gadopiclenol and gadoquatrane – have relaxivities^26,27^ even close to that of GdDOTBA at 310 K and 1.5 or 3.0 T. However, care is required when comparing these two agents with GdDOTBA or any of the established clinical agents. Gadopiclenol employs a heptadentate ligand opening a second coordination site to water, a strategy that was once considered risky for a chelate that is to be used *in vivo* because of the risk of compromising chelate robustness. This chelate does not meet the criteria for Fig. 1. Gadoquatrane increases the number of Gd^3+^ ions from 1 to 4, making the agent significantly larger (a 2.4-fold increase in molecular weight over GdDOTBA). Fig. 4 (right) shows relaxivity per Gd^3+^ coordinated water molecule – which provides a better assessment of the ability of the Gd^3+^ ion in each agent to relax solvent water protons. It is clear from Fig. 4 (right) that GdDOTBA is an especially good relaxation agent with the potential to reduce doses by a factor of two or more even at high fields.

**Fig. 4:**
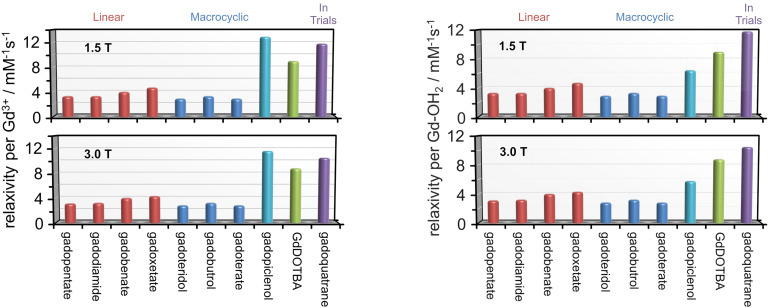
Left: The relaxivity (expressed in terms of the relaxivity per Gd^3+^) of GdDOTBA at 1.5 T (top) and 3.0 T (bottom) at 310 K. The relaxivities of clinically available contrast agents and another currently in trials under the same conditions are shown for comparative purposes. Right: The relaxivity (expressed in terms of the relaxivity per water molecule bound to Gd^3+^) of GdDOTBA at 1.5 T (top) and 3.0 T (bottom) at 310 K. The relaxivities of clinically available contrast agents and another currently in trials under the same conditions are shown for comparative purposes.

α-Aryl substituted GdDOTA derivatives can afford the high relaxivities that may allow the same contrast to be generated with lower doses. But are these chelates robust enough to meet the increasingly stringent safety requirements for *in vivo* use? As a preliminary investigation, GdDOTFA was incubated at 0.42 mM in 1 M HCl at 298 K and dissociation of the metal ion from the chelate monitored by relaxometry. After 216 hours of incubation, no detectable change in the water proton *R*_1_ could be measured (Fig. S6, ESI†). Under the same conditions, Gd^3+^ was found to dissociate for DOTA with a half-life measured in hours. This implies that α-aryl substituted GdDOTA chelates are not just robust enough for *in vivo* use but may be some of the most robust Gd^3+^ chelates yet developed.

In conclusion, after decades of research α-aryl substituted GdDOTA chelates may represent the optimal solution for designing of contrast agents for MRI. Previous efforts have shown that one, or even several, of the key properties of Gd^3+^ chelates that affect relaxivity can be optimized. To our knowledge α-aryl substituted GdDOTA chelates are the only system that simultaneously optimize all of them. The proportion of TSAP isomer is increased leading to rapid water exchange kinetics. The symmetrical ligand field is shielded from modulation by collision, improving the electronic relaxation. The aryl substituents permit binding to macromolecules that slows chelate tumbling. Furthermore, the synthesis of these chelates is straightforward.^19^

GdDOTFA, when bound to the HSA, is the first monohydrated chelate to afford the peak relaxivity at 0.5 T and 298 K. GdDOTBA, as a discrete chelate, exhibits unprecedentedly high relaxivity even at the higher fields (1.5 T and 3.0 T) typically used in clinical MRI. In addition to their outstanding performance as relaxation agents, these chelates are found to be exceptionally robust to chelate dissociation. α-Aryl substitution may prove to be the key to achieving the safest, most effective contrast agents for MRI.^10^

Conceptualization: KBM, MB, MW; investigation: KBM, LNR, FC; data curation and formal analysis: all authors; writing: all authors; funding acquisition: KBM, MB, MW; project administration: MW.

The authors thank the National Institutes of Health (GM-127964, EB-034082, MW), the National Science Foundation (034082, KBM) (MRI 1828573) and Ministero dell’Università e della Ricerca (PRIN 2017A2KEPL) (MB) for financial support.

## Conflicts of interest

There are no conflicts to declare.

## Supplementary Material

CC-060-D3CC05989H-s001
