# Supplementary material for: α-Aryl substituted GdDOTA derivatives, the perfect contrast agents for MRI?
Source: Chem Commun (Camb). 2024 Jan 9;60(21):2898–901. doi: 10.1039/d3cc05989h (PMC10919327; doi:10.1039/d3cc05989h)
Supplement: CC-060-D3CC05989H-s001 [file CC-060-D3CC05989H-s001.pdf]

Electronic Supplementary Material (ESI) for ChemComm.
This journal is © The Royal Society of Chemistry 2024

Electronic Supplementary Information

for

α-Aryl Substituted GdDOTA Derivatives, the Perfect Contrast Agents for MRI?

Karley B. Maier[a] *0000-0001-8573-2873*

Lauren N. Rust[a] *0000-0001-8585-466X*

Fabio Carniato[b] *0000-0002-6268-1687*

Mauro Botta[b] *0000-0003-4192-355X*

Mark Woods[a,c] *0000-0003-4890-7544*

[a] Department of Chemistry, Portland State University, 1719 SW 10th Ave, Portland OR, 97201, USA

[b] Departimento di Scienze e Innovazione Tecnolgica, Università del Peimonte Orientale "Amedeo Avogadro", I-15121 Alessandria, Italy

[c] Advanced Imaging Research Center, Oregon Health and Science University, 1381 SW Sam Jackson Park Road, Portland OR, 97239, USA

E-mail: mark.woods@pdx.edu or mauro.botta@uniupo.it

Table of Contents

Figure S1. **S3**

The temperature dependence of the ^{17}O NMR reduced transverse relaxation rate constant of solvent water of solutions of GdDOTFA (left) and GdDOTBA (right) at pH 6.6 and 11.7 T.

Figure S2. **S3**

Left: Relaxometric titration of human serum albumin into a 0.09 mM solution of GdDOTFA at 298 K and 0.47 T (red circles). For comparative purposes the effect on the relaxation rate constant of HSA (black diamonds) and GdDOTFA alone (open diamonds) are shown. Right: Relaxometric titration of human serum albumin into a 0.10 mM solution of GdDOBTA at 298 K and 0.47 T.

Figure S3. **S4**

The NMRD profile of GdDOTFA in human serum (seronorm) recorded at 310 K and a total [Gd] = 0.5 mM (open red diamonds). The ordinate axis is not expressed in terms of relaxivity, but in terms of the paramagnetic effect on the relaxation rate constant of $^{1}H_2O$ without correction for concentration. For comparative purposes the NMRD profile of GdDOTFA in aqueous solution at 310 K at the same concentration is shown (closed blue diamonds). The concentration of GdDOTFA bound to HSA in serum is estimated to be in the range of 50 μM.

Figure S4. **S5**

The structures of Gd^{3+} chelates used as contrast agents that are either in clinical trials or already in clinical use. The relaxivities of these agents are shown in Figures 4 & 5. Coordinated water molecules are not shown, but these chelates are coordinatively saturated when CN=9, the maximum number of coordinated water molecules can therefore be calculated by subtracting the denticity of the ligand from 9.

Figure S5. **S6**

The calculated relaxivity, as function of the water exchange lifetime (τ_M), of a Gd^{3+} chelate with electronic relaxation characteristics comparable to those of GdDOTA (Δ^2 = 1.6 × 10^{-19} s^{-2}, τ_V = 7.7 ps) if the rate of molecular tumbled had been slowed such that τ_R =100 ns (left) and τ_R = 2 ns (right) at different magnetic field strengths. When τ_R is long, the value of τ_M is shorter at higher magnetic fields.

Figure S6. **S6**

The calculated relaxivity, as function of the water exchange lifetime (τ_M), of a Gd^{3+} chelate with electronic relaxation characteristics comparable to those of GdDOTFA (Δ^2 = 6.8 × 10^{-18} s^{-2}, τ_V = 25 ps) if the rate of molecular tumbled had been slowed such that τ_R =100 ns (left) and τ_R =

2 ns (right) at different magnetic field strengths. The significance of optimizing electronic relaxation is evident when comparing with the relaxivity that achieved in Figure S3.

Figure S7. **S7**

The water proton relaxation rate constant (R_1) measured during incubation of a 0.42 mM solution of GdDOTFA (blue open circles) and a 0.46 mM solution of GdDOTA (black triangles) in 1M HCl at 298 K. Measured at 298 K and 32 MHz.

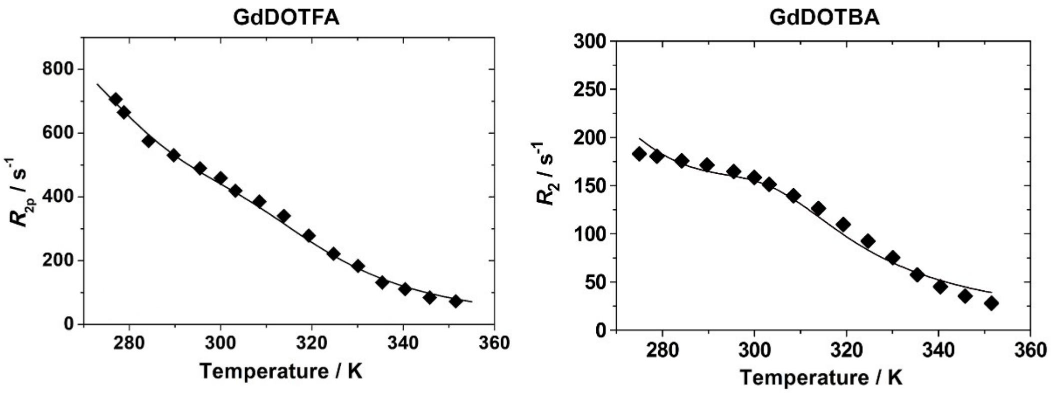

Figure S1. The temperature dependence of the ^{17}O NMR reduced transverse relaxation rate constant of solvent water of solutions of GdDOTFA (left) and GdDOTBA (right) at pH 6.6 and 11.7 T.

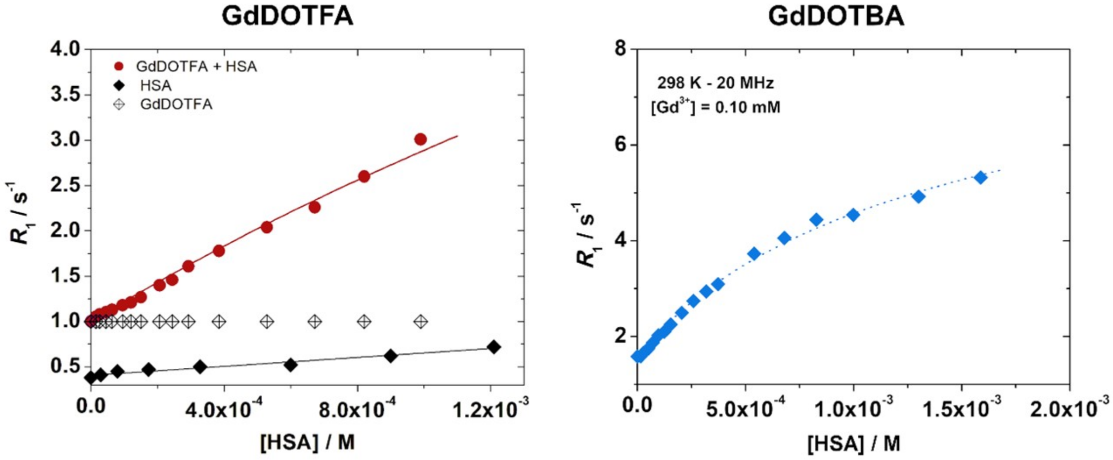

Figure S2. Left: Relaxometric titration of human serum albumin into a 0.09 mM solution of GdDOTFA at 298 K and 0.47 T (red circles). For comparative purposes the effect on the relaxation rate constant of HSA (black diamonds) and GdDOTFA alone (open diamonds) are shown. Right: Relaxometric titration of human serum albumin into a 0.10 mM solution of GdDOBTA at 298 K and 0.47 T.

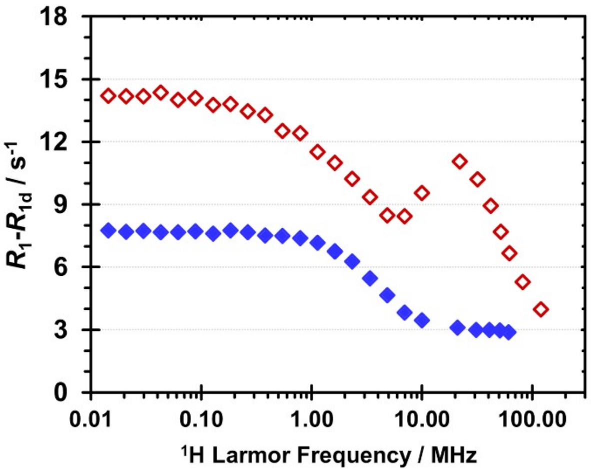

Figure S3. The NMRD profile of GdDOTFA in human serum (seronorm) recorded at 310 K and a total [Gd] = 0.5 mM (open red diamonds). The ordinate axis is not expressed in terms of relaxivity, but in terms of the paramagnetic effect on the relaxation rate constant of ^1H_2O without correction for concentration. For comparative purposes the NMRD profile of GdDOTFA in aqueous solution at 310 K at the same concentration is shown (closed blue diamonds). The concentration of GdDOTFA bound to HSA in serum is estimated to be in the range of 50 μM.

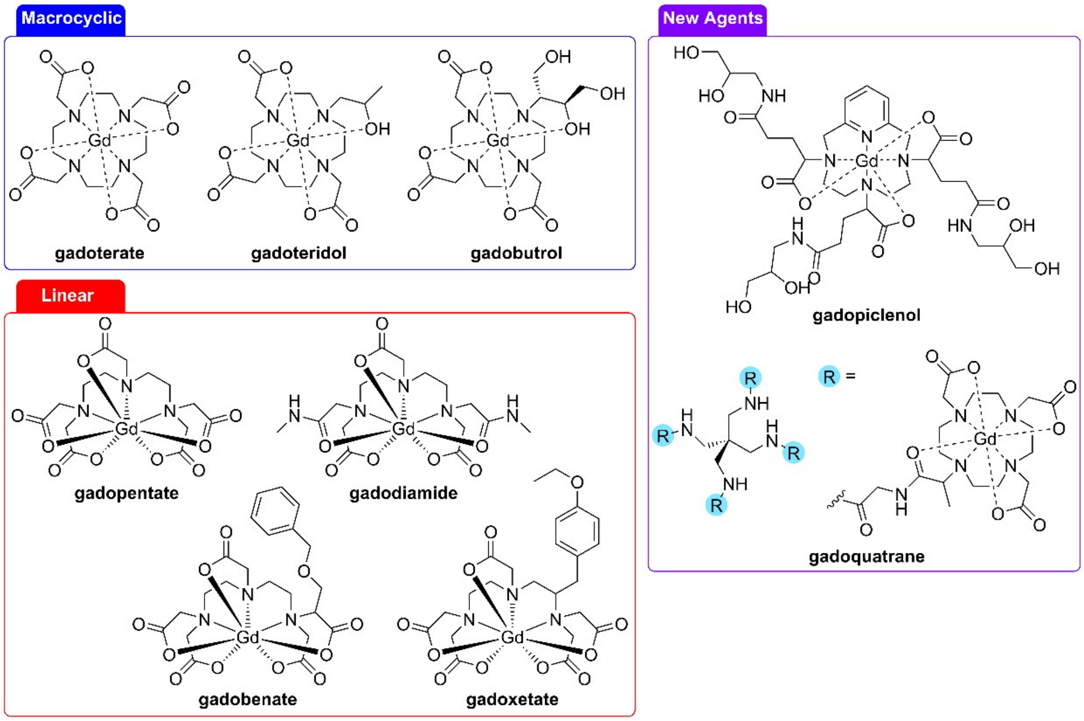

Figure S4. The structures of Gd^{3+} chelates used as contrast agents that are either in clinical trials or already in clinical use. The relaxivities of these agents are shown in Figures 4 & 5. Coordinated water molecules are not shown, but these chelates are coordinatively saturated when CN=9, the maximum number of coordinated water molecules can therefore be calculated by subtracting the denticity of the ligand from 9.

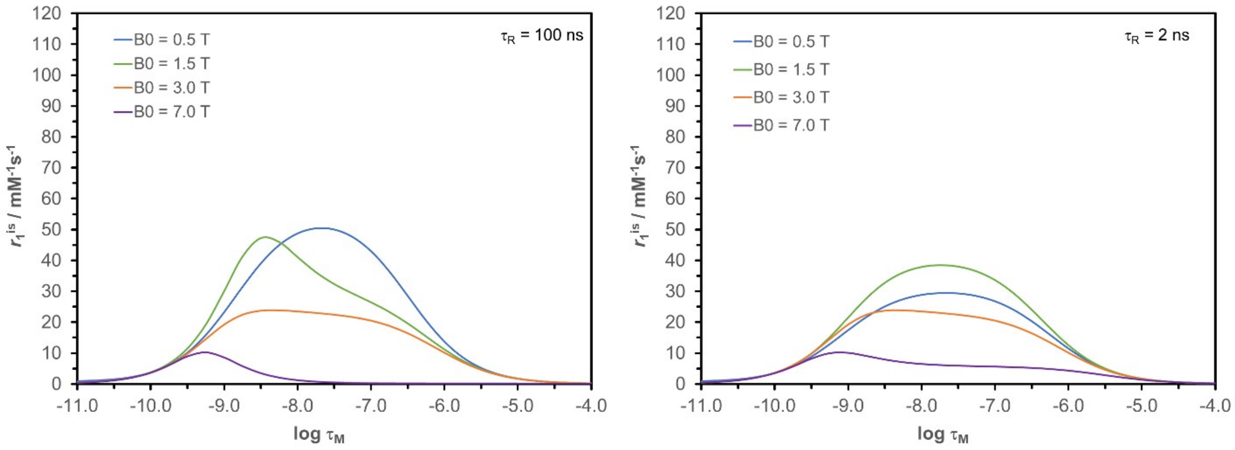

Figure S5. The calculated relaxivity, as function of the water exchange lifetime (τ_M), of a Gd^{3+} chelate with electronic relaxation characteristics comparable to those of GdDOTA ($\Delta^2 = 1.6 \times 10^{-19}$ s^{-2}, τ_V = 7.7 ps) if the rate of molecular tumbled had been slowed such that τ_R =100 ns (left) and τ_R = 2 ns (right) at different magnetic field strengths. When τ_R is long, the value of τ_M is shorter at higher magnetic fields.

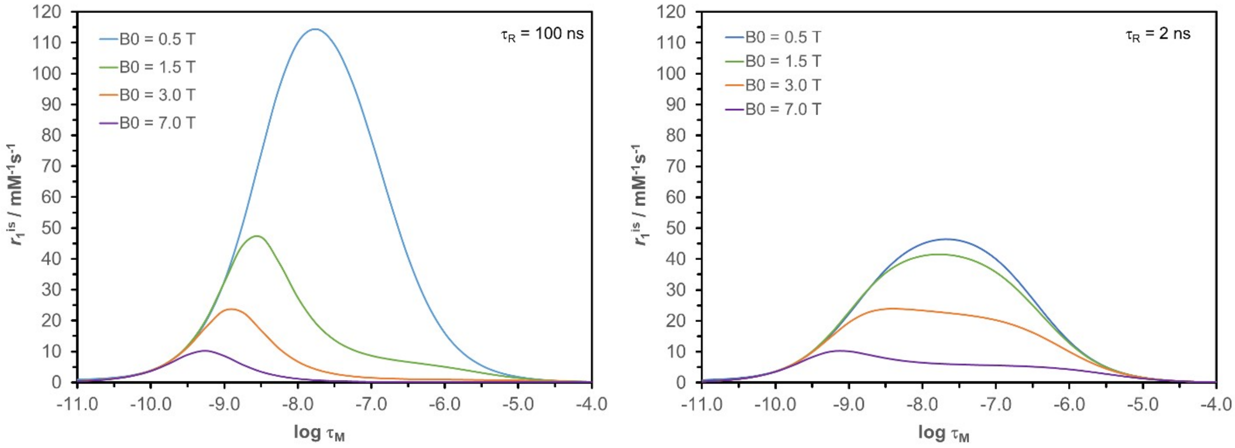

Figure S6. The calculated relaxivity, as function of the water exchange lifetime (τ_M), of a Gd^{3+} chelate with electronic relaxation characteristics comparable to those of GdDOTFA ($\Delta^2 = 6.8 \times 10^{-18}$ s^{-2}, τ_V = 25 ps) if the rate of molecular tumbled had been slowed such that τ_R =100 ns (left) and τ_R = 2 ns (right) at different magnetic field strengths. The significance of optimizing electronic relaxation is evident when comparing with the relaxivity that achieved in Figure S3.

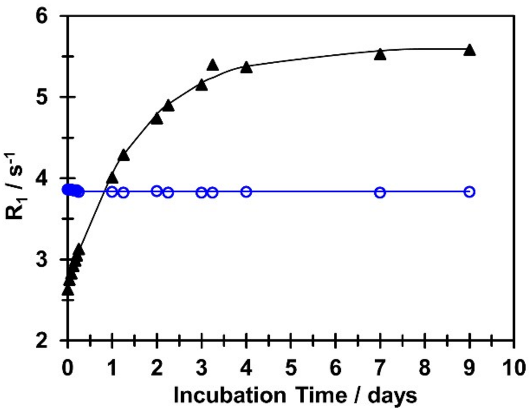

Figure S7. The water proton relaxation rate constant (R_1) measured during incubation of a 0.42 mM solution of GdDOTFA (blue open circles) and a 0.46 mM solution of GdDOTA (black triangles) in 1M HCl at 298K. Measured at 298K and 32 MHz.
